# Lipid Metabolism Is Dysregulated in the Motor Cortex White Matter in Amyotrophic Lateral Sclerosis

**DOI:** 10.3390/metabo12060554

**Published:** 2022-06-17

**Authors:** Gemma L. Sadler, Katherine N. Lewis, Vinod K. Narayana, David P. De Souza, Joel Mason, Catriona McLean, David G. Gonsalvez, Bradley J. Turner, Samantha K. Barton

**Affiliations:** 1 Florey Institute of Neuroscience and Mental Health, Melbourne 3052, Australia; gsadler1@student.unimelb.edu.au (G.L.S.); klewis3@student.unimelb.edu.au (K.N.L.); joel.mason@florey.edu.au (J.M.); bradley.turner@florey.edu.au (B.J.T.); 2 Metabolomics Australia, Bio21 Institute, University of Melbourne, Melbourne 3052, Australia; vinod.narayana@unimelb.edu.au (V.K.N.); desouzad@unimelb.edu.au (D.P.D.S.); 3 Victorian Brain Bank, Florey Institute of Neuroscience and Mental Health, Melbourne 3052, Australia; catriona.mclean@monash.edu; 4 Department of Anatomy and Developmental Biology, Monash University, Melbourne 3168, Australia; david.gonsalvez@monash.edu.au

**Keywords:** ALS, myelin, lipid metabolism

## Abstract

Lipid metabolism is profoundly dysregulated in amyotrophic lateral sclerosis (ALS), yet the lipid composition of the white matter, where the myelinated axons of motor neurons are located, remains uncharacterised. We aimed to comprehensively characterise how myelin is altered in ALS by assessing its lipid and protein composition. We isolated white matter from the motor cortex from post-mortem tissue of ALS patients (n = 8 sporadic ALS cases and n = 6 familial ALS cases) and age- and sex-matched controls (n = 8) and conducted targeted lipidomic analyses, qPCR for gene expression of relevant lipid metabolising enzymes and Western blotting for myelin proteins. We also quantified myelin density by using spectral confocal reflectance microscopy (SCoRe). Whilst myelin protein composition was similar in ALS and control tissue, both the lipid levels and the expression of their corresponding enzymes were dysregulated, highlighting altered lipid metabolism in the white matter as well as a likely change in myelin composition. Altered myelin composition could contribute to motor neuron dysfunction, and this highlights how oligodendrocytes may play a critical role in ALS pathogenesis.

## 1. Introduction

Amyotrophic lateral sclerosis (ALS) patients have systemic defects in their energy metabolism and commonly have hypolipidemia with a lower body mass index being associated with a higher risk of developing ALS [1]. A small double-blind phase II trial of ALS patients found those who consumed a high-fat and high-caloric diet survived longer than patients who consumed a regular diet [2]. However, the precise molecular mechanisms underpinning exactly how energy supplementation is beneficial in ALS remain unknown. Glia are extremely metabolically active cells in the central nervous system, yet they remain relatively unexplored in regard to their contribution to neuronal metabolic failure. Oligodendrocytes, in particular, have high surface area contact with neurons through their myelin and are responsible for metabolically supporting neuronal health and function, as well as maintaining the rapid conduction velocity of neurons [3,4,5,6]. These functions rely on tight control during myelin development and maintenance. TAR DNA-binding protein 43 (TDP-43), which pathologically accumulates in 97% of ALS patients (including in oligodendrocytes [7]), has been shown to be integral to normal oligodendrocyte function and myelin production [8], thereby raising the question as to how pathogenic TDP-43 in ALS may impact on normal oligodendrocyte function. Indeed, oligodendrocytes have been found to have a cell-autonomous deficit in ALS [9,10,11,12,13], yet their precise role in ALS certainly requires further characterisation. Myelin can respond dynamically to changes in neuronal activity [14,15], yet oligodendrocytes are diseased in ALS and are required to meet high metabolic demands. Thus, it seems likely that there could be fundamental changes in oligodendrocyte function and myelin composition in ALS.

Lipids account for 50–60% of the brain’s dry weight with a substantial proportion of these lipids existing in myelin. Indeed, myelin is composed of 70–80% lipids [16]. Unfortunately, there are no myelin-specific lipids, which renders their characterisation difficult. It has been suggested that the lipid signature of the white matter is reflective of myelin composition and grey matter composition is significantly different from that of myelin [16]. To date, lipidomic assessment in ALS is limited to the grey matter [17] (or to CSF [18]), and remains under-characterised in the motor cortex and certainly remains unexplored in the white matter. The remaining 20–30% of myelin is composed of protein. In ALS, the assessment of myelin protein composition has been in superoxide dismutase (SOD1) mutant mice and human post-mortem tissue, but is mostly limited to myelin basic protein (MBP) and proteolipid protein (PLP) [10,12,13,19]. Further, the characterisation of these proteins has produced variable results, such that the precise protein composition of myelin in ALS remains unclear. Finally, myelin analyses are predominantly performed in the lumbar spinal cord where neuronal loss is high, which would skew findings. By assaying the motor cortex, we are more likely to identify myelin changes that can, at least in part, be attributed intrinsically to the oligodendrocytes.

Myelin characterisation in ALS lacks the depth required to truly appreciate its changes at a molecular level. This is partly due to the challenges faced in studying myelin due to the complex relationship between myelin and its ensheathed axon, rendering it difficult to determine the source of change. However, given the recent advancements regarding the involvement of oligodendrocytes in ALS pathogenesis [8,9,11,20], coupled with the importance of myelin for normal neuronal functioning, it is imperative that myelin be appropriately characterised in ALS. We aimed to more extensively characterise changes in myelin by focusing on the underappreciated white matter region of the motor cortex using human post-mortem tissue. By combining targeted lipidomic analyses, protein analyses by Western blotting and myelin imaging using spectral confocal reflectance (SCoRe) microscopy, we are the first to comprehensively assess myelin composition in ALS.

## 2. Results

### 2.1. ALS Patients Have an Altered Metabolic Signature in Their Motor Cortex White Matter

**Figure 1:**
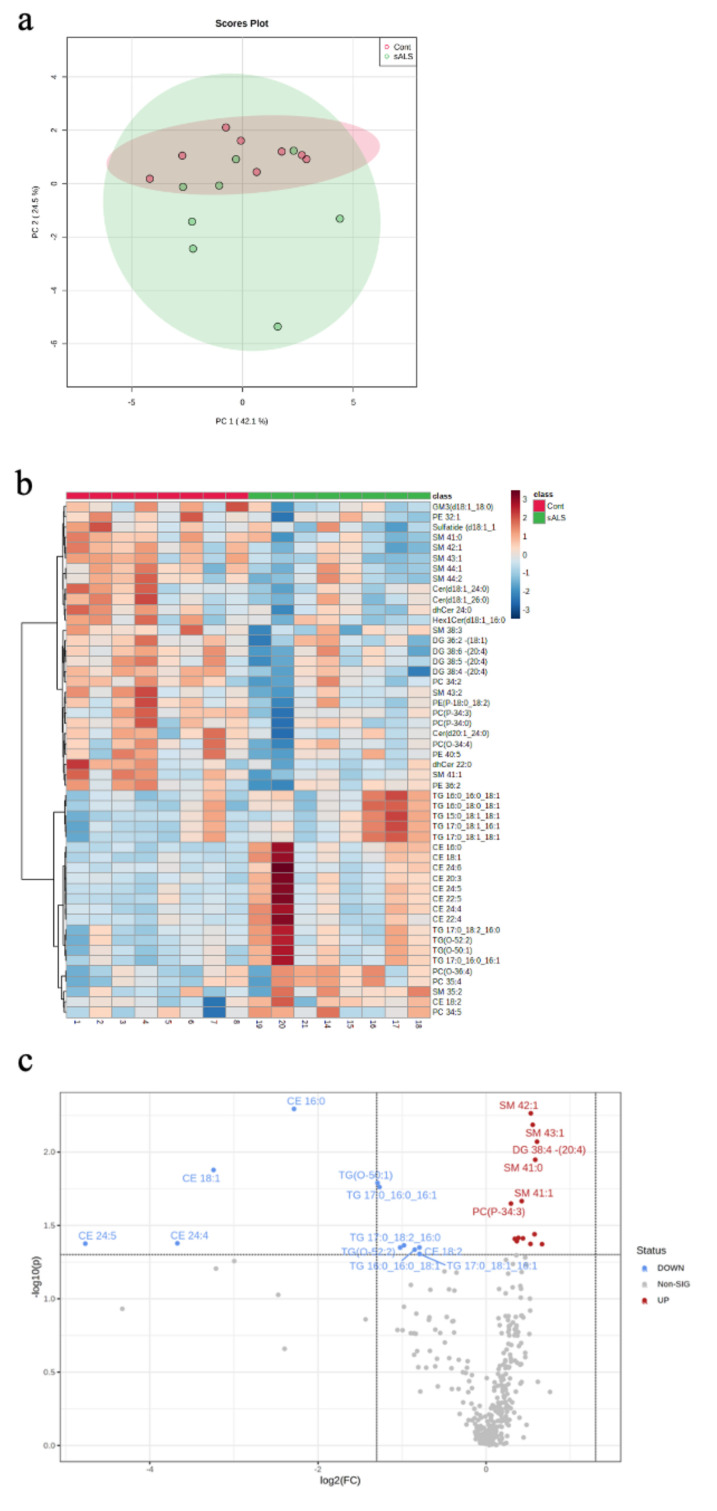
Targeted lipidomic analyses of motor cortex white matter in ALS versus control. (**a**) A PCA plot depicting separation of ALS samples (sALS; n = 8) from age- and sex-matched controls (n = 8); (**b**) a heatmap highlighting the 50 most differentially expressed lipid species grouped as control (pink) and sALS (green) (above heatmap) identified by case number (below heatmap; see Table 1); (**c**) a volcano plot highlighting the lipid species that are significantly increased (blue) and decreased (red) in sALS compared to control.

Targeted lipidomic profiling was carried out on white matter dissected from the motor cortex of sporadic ALS cases (sALS; n = 8) and age- and sex-matched controls (control; n = 8). Unsupervised grouping of cases using principal component analysis (PCA; PC = 2, R^2^X = 0.42, R^2^Y = 0.25; Figure 1a) showed high variance in the ALS cases. The heatmap visualisation of the top 50 differentially abundant lipid species reflected this variability, whilst also showing a distinct separation between ALS and control (Figure 1b). A volcano plot analysis highlighted 24 significantly different lipid species and 311 non-significant lipids (Figure 1c); the lipid species that were significantly higher in control compared to ALS are shown in red and those that are significantly lower in control than ALS are shown in blue. To further explain the separation in the lipidomic profiles between ALS and control, a partial least squares-discriminant analysis (PLS-DA) was performed (PC = 2, R^2^X = 0.24, R^2^Y = 0.094; Supplementary Materials, Figure S1a). A variable importance in projection (VIP) plot showed the 25 most important metabolites identified by the PLS-DA that contribute to this separation (Supplementary Materials, Figure S1b), which predominantly include cholesterol esters and triacylglycerides.

**Figure 2:**
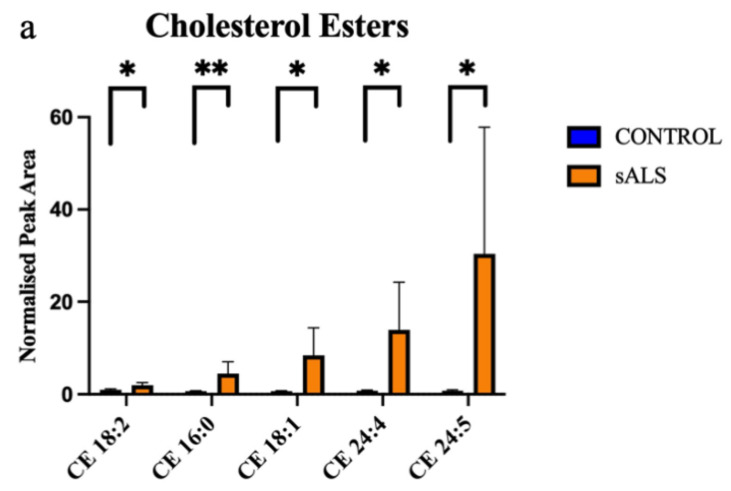

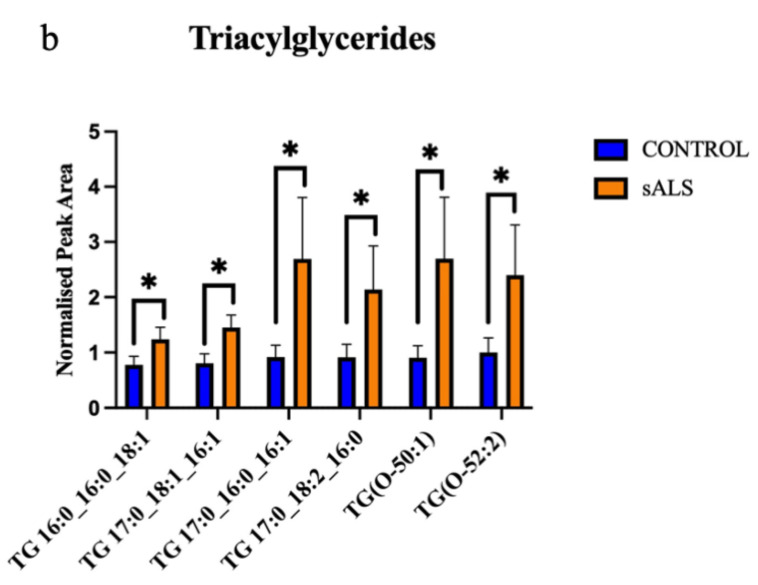
Cholesterol esters and triacylglycerides are increased in ALS compared to control in the motor cortex white matter. (**a**) Cholesterol esters (CE) that have a significantly increased normalised peak area in ALS (orange; sALS n = 8) compared to control (blue; n = 8); (**b**) triacylglycerides (TG) that have a significantly increased normalised peak area in ALS (orange; sALS n = 8) compared to control (blue; n = 8). * *p* < 0.05; ** *p* < 0.01.

**Figure 3:**
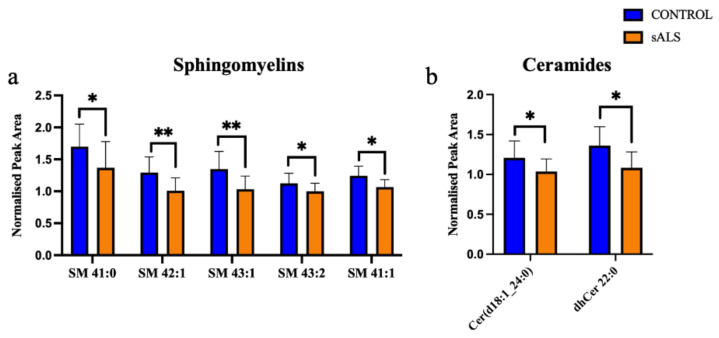

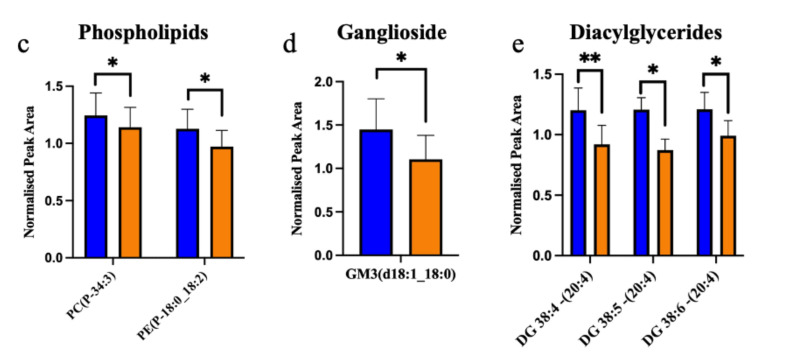
Ceramides, glycerolipid, phospholipids and sphingomyelin are decreased in ALS compared to control in the motor cortex white matter. (**a**) Sphingomyelins (SM); (**b**) ceramides (Cer); (**c**) phosphatidylcholine (PC) and phosphatidylethanolamine (PE); (**d**) ganglioside (GM); (**e**) diacylglycerides (DG) have a significantly decreased normalised peak area in ALS (orange; sALS n = 8) compared to control (blue; n = 8); * *p* < 0.05; ** *p* < 0.01.

Of the 24 significantly changed lipid species, 11 were significantly increased in the white matter of the motor cortex in ALS compared to control. These increased lipids were cholesterol esters (Figure 2a) and triacylglycerides (Figure 2b). The remaining 13 significantly altered lipid species were downregulated in ALS cases, compared to control, and comprised 5 sphingomyelins (Figure 3a), 2 ceramides (Figure 3b), 2 phospholipids (Figure 3c), 1 ganglioside (Figure 3d) and 3 diacylglycerides (Figure 3e).

### 2.2. The Enzymes Responsible for Lipid Metabolism Are Also Altered in the Motor Cortex White Matter of ALS Patients

**Figure 4:**
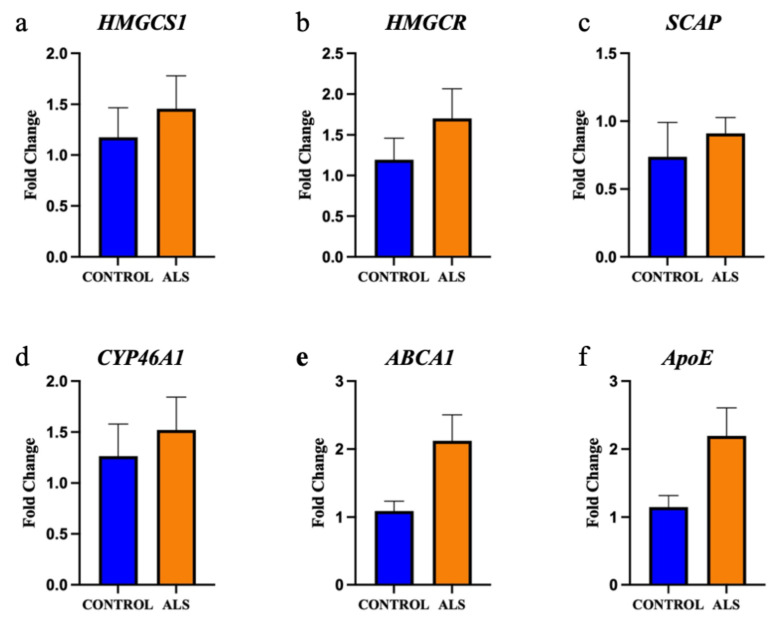
The gene expression of key enzymes involved in cholesterol metabolism are not altered in the motor cortex white matter in ALS. The gene expression of the enzymes: (**a**) hydroxymethylglutaryl-CoA synthase (*HMGCS1*); (**b**) HMG-CoA reductase (*HMGCR*); (**c**) SREBP cleavage-activating protein (*SCAP*); (**d**) cholesterol 24-hydroxylase (*CYP46A1*); (**e**) ATP-binding cassette transporter A1 (*ABCA1*); (f) Apolipoprotein E (*ApoE*) are not significantly different in the motor cortex white matter of ALS (sALS n = 8 and fALS n = 6 combined; orange) and control (n = 8; blue) cases.

**Figure 5:**
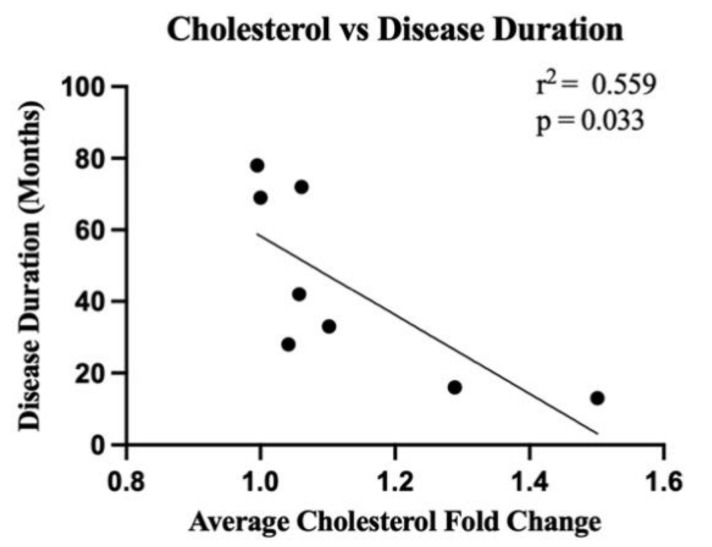
There is a significant correlation between cholesterol levels in the white matter of the motor cortex in ALS cases and disease duration. Linear regression analyses highlight the correlation (r^2^ = 0.559) between the average of all cholesterol lipid species in ALS cases (sALS n = 8; expressed as a fold change from the average of all controls (n = 8)) and disease duration (months) (*p* = 0.033).

The gene expression of key lipid metabolising enzymes was measured using qRT-PCR and compared between ALS (n = 8 sporadic ALS cases and n = 6 familial ALS cases combined) and control (n = 8); the relationship between the genes (and translated enzymes) and the associated lipids is schematically presented in Supplementary Materials, Figure S2. Upstream of cholesterol synthesis, hydroxymethylglutaryl-CoA synthase (HMGCS1) metabolises acetyl-CoA to HMG-CoA, which is further metabolised to mevalonic acid via HMG-CoA reductase (HMGCR); neither *HMGCS1* (Figure 4a; *p* = 0.59) nor *HMGCR* (Figure 4b; *p* = 0.35) were expressed differently between groups. SREBP cleavage-activating protein (SCAP) feeds back to control cholesterol synthesis by regulating HMG-CoA reductase levels; *SCAP* was also not differentially abundant between groups (Figure 4c; *p* = 0.48). The enzyme responsible for the majority of cholesterol turnover in the central nervous system is cholesterol 24-hydroxylase (CYP46A1); *CYP46A1* levels were also not different between groups (Figure 4d; *p* = 0.62). Two transporters known to assist in cholesterol transport are the ATP-binding cassette transporter A1 (*ABCA1*; Figure 4e; *p* = 0.061) and apolipoprotein E (*ApoE;* Figure 4f; *p* = 0.078), which were also similar in ALS and control groups. We next wanted to characterise the correlation between metabolic enzyme gene expression and total lipid expression and to assess whether these correlations differed in ALS patients compared to control, as a representation of altered capacity to metabolise lipids. There was no significant difference in correlation between cholesterol and *HMGCS1*, *HMGCR*, *SCAP*, *CYP46A1*, *ABCA1* or *ApoE* (Supplementary Materials, Figure S3a–f); however, when qualitatively assessing these data, it could be seen that whilst the control patients had tight regulation over their cholesterol levels, there was much higher variability in the sALS patients. When comparing the correlation between cholesterol levels in ALS patients and disease duration, it was found that higher cholesterol levels in the white matter of the motor cortex were correlated with shorter disease duration (Figure 5; r^2^ = 0.559; *p* = 0.033).

**Figure 6:**
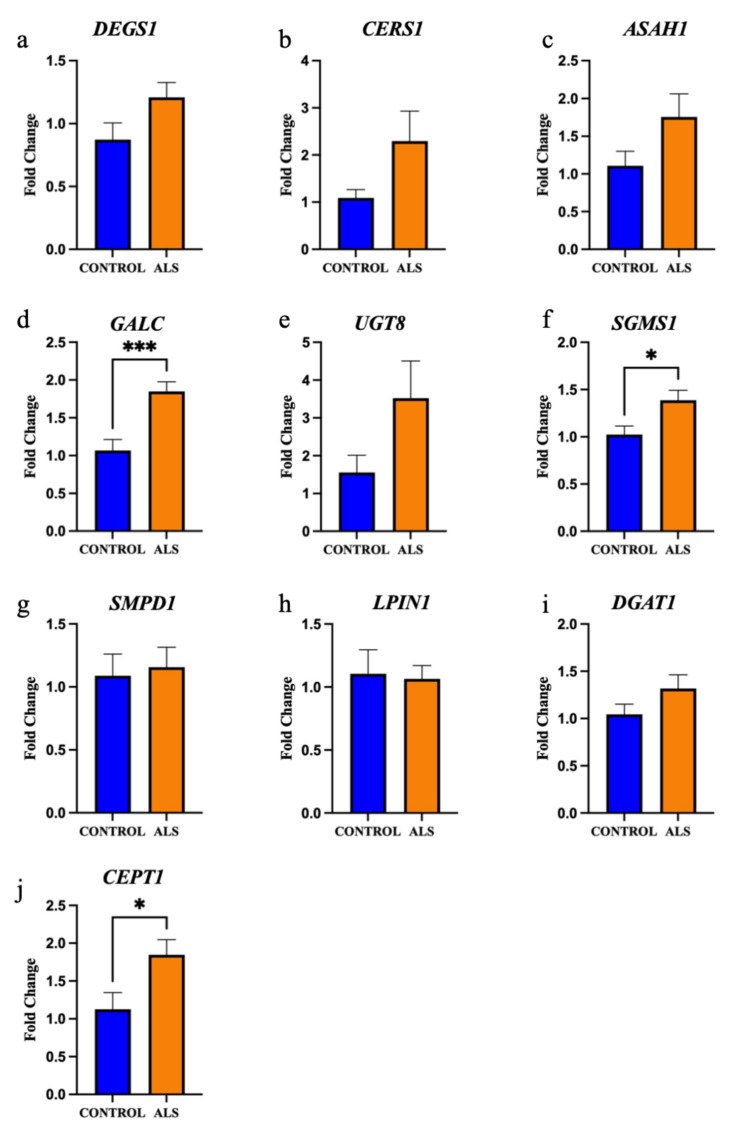
The gene expression of key enzymes involved in ceramide and diacylglyceride metabolism are significantly increased in the motor cortex white matter in ALS. The gene expression of the enzymes: (**a**) sphingolipid desaturase (*DEGS1*); (**b**) ceramide synthase 1 (*CERS1*); (**c**) acid ceramidase (*ASAH1*); (**d**) galactosylceramidase (*GALC*); (**e**) ceramide galactosyltransferase (*UGT8*); (**f**) sphingomyelin synthase 1 (*SGMS1*); (**g**) acid sphingomyelinase (SMase, encoded by *SMPD1*); (**h**) phosphatidate phosphatase (*LPIN1*); (**i**) diacylglycerol O-acyltransferase 1 (*DGAT1*); and (**j**) choline/ethanolamine phosphotransferase 1 (*CEPT1*) expressed as a fold change in ALS (sALS n = 8, fALS n = 6 combined; orange) compared to control (n = 8; blue). * *p* < 0.05 *** *p* < 0.001.

We next focused on ceramide metabolism. Sphingolipid desaturase (DEGS1) is responsible for the final step in the metabolism of dihydroceramide to ceramide; *DEGS1* was not differentially expressed between groups (Figure 6a; *p* = 0.09). Whilst there was a positive correlation between ceramide lipid levels and *DEGS1* expression in control cases (r = 0.706), the correlation was negative in ALS cases (r = −0.650) and the difference between these correlations was significantly different (Supplementary Materials, Figure S4a; *p* = 0.016). This same relationship was observed between sphingomyelin levels and *DEGS1* expression, but it did not reach statistical significance (r = 0.619 and r = −0.492, respectively; *p* = 0.064; Supplementary Materials, Figure S4c). Ceramide synthase 1 (CERS1); and acid ceramidase (ASAH1) are involved in the metabolism of ceramide to sphingosine; *CERS1* (Figure 6b; *p* = 0.16) and *ASAH1* (Figure 6c; *p* = 0.14) levels were not different between groups. Galactosylceramidase (GALC) and ceramide galactosyltransferase (UGT8) are involved in the metabolism of ceramide to galactosylceramide; *GALC* expression was significantly higher in ALS compared to control (Figure 6d; *p* = 0.0009), but there was no difference in *UGT8* expression (Figure 6e; *p* = 0.16). Sphingomyelin synthase 1 (SGMS1) and acid sphingomyelinase (SMase) are involved in the metabolism of ceramide to sphingomyelin; *SGMS1* expression was significantly higher in ALS compared to control (Figure 6f; *p* = 0.03), but there was no difference in *SMPD1* (encodes SMase; Figure 6g; *p* = 0.78) expression. There was a positive correlation between ceramide levels and *SMPD1* levels in controls (r = 0.658), but a negative correlation in ALS cases (r = −0.665) and these correlations reached statistical difference (Supplementary Materials, Figure S4b; *p* = 0.015). There was also a positive correlation between sphingomyelin levels and *SMPD1* levels in controls (r = 0.756), but a negative correlation in ALS cases (r = −0.673) and this also reached statistical significance (*p* = 0.008; Supplementary Materials, Figure S4d).

Next, we focused on diacylglyceride metabolism. Phosphatidate phosphatase (LPIN1) and diacylglycerol O-acyltransferase 1 (DGAT1) are involved in the metabolism of diacylglyceride to triacylglyceride; neither *LPIN1* (Figure 6h; *p* = 0.84) nor *DGAT1* (Figure 6i; *p* = 0.21) expression were different between groups. Choline/ethanolamine phosphotransferase 1 (CEPT1) is involved in the metabolism of diacylglyceride to phosphatidylcholine and phosphatidylethanolamine; *CEPT1* was significantly higher in ALS cases compared to control (Figure 6j; *p* = 0.046).

### 2.3. The Expression of Myelin Proteins Was Not Altered in the White Matter of the Motor Cortex in ALS

**Figure 7:**
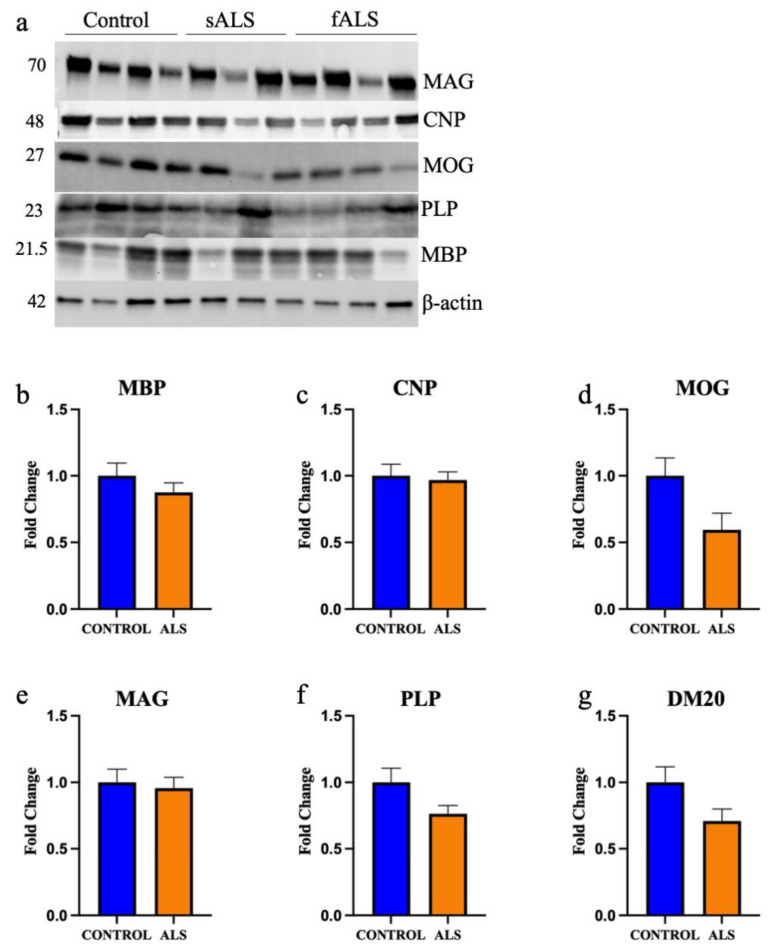
The protein expression of key myelin proteins is not altered in the motor cortex white matter in ALS. (**a**) A representative blot of key myelin proteins measured using Western blot in sALS, fALS and control. Numbers denote protein size in kDa. The protein expression of (**b**) myelin basic protein (MBP); (**c**) 2′,3′-cyclic-nucleotide 3′phosphodiesterase (CNPase); (**d**) myelin oligodendrocyte glycoprotein (MOG); (**e**) myelin-associated glycoprotein (MAG); (**f**) proteolipid protein (PLP); and (**g**) DM-20 are not different in ALS (sALS n = 8 and fALS n = 6 combined; orange) compared to control (n = 8).

We next carried out Western blot analyses of key myelin proteins in the motor cortex white matter (Figure 7a) in ALS cases (sALS n = 8 and fALS n = 6 combined) and controls (n = 8). There was no difference in MBP (Figure 7b; *p* = 0.31), 2’,3’-cyclic-nucleotide 3’-phosphodiesterase (CNPase; Figure 7c; *p* = 0.76), myelin oligodendrocyte glycoprotein (MOG; Figure 7d; *p* = 0.051), myelin associated glycoprotein (MAG; Figure 7e; *p* = 0.74), PLP (Figure 7f; *p* = 0.054) or DM20 (Figure 7g; *p* = 0.065) levels. There was also no difference in the proportion of OLIG2-positive oligodendrocytes in the motor cortex white matter (Supplementary Materials, Figure S5; *p* = 0.52).

### 2.4. SCoRe Microscopy Shows No Change to Myelin Density in the White Matter of the Motor Cortex in ALS

**Figure 8:**
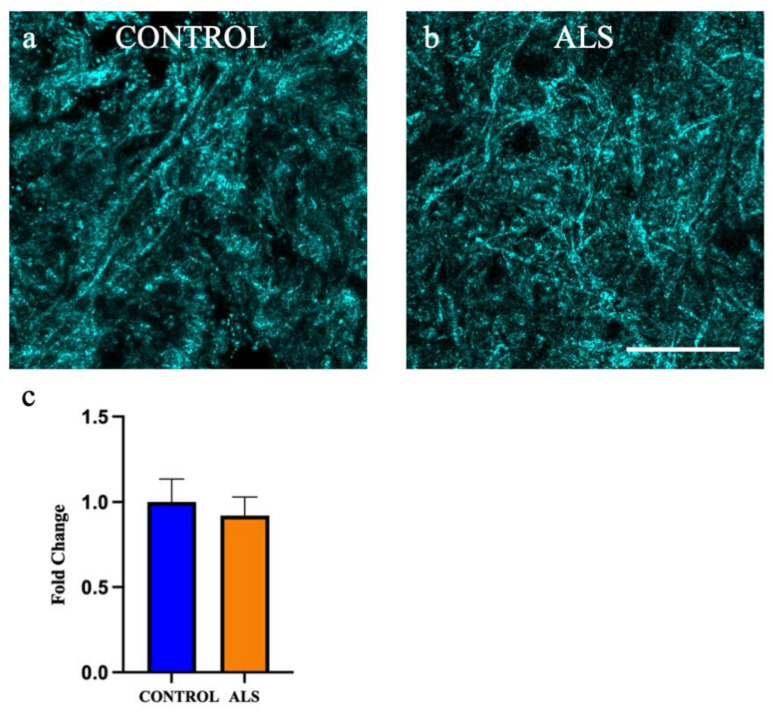
Myelin density is not altered in the white matter of the motor cortex in ALS. Representative images of the white matter of the motor cortex taken using spectral reflectance microscopy (SCoRe) in the (**a**) control and (**b**) sALS case. (**c**) there was no difference in the amount of myelin in the white matter of the motor cortex in ALS (sALS n = 8; orange) when presented as a fold change from control (n = 8; blue). Scale bar 25 µm.

Ascertaining the absolute amount of myelin remains challenging given the complexity of its composition, as well as the harshness of the techniques required to visualise myelin, such as antigen retrieval. We chose to utilise spectral confocal reflectance (SCoRe) microscopy, which allows high resolution imaging of compact myelin through the quantification of endogenous reflected light off the lipid portion of myelin. Extensive compact myelin could be seen in the white matter of the motor cortex in all control cases (Figure 8a shows a representative image of a control case) and all ALS cases (Figure 8b shows a representative image of an sALS case). There was no difference in the amount of myelin in the white matter of the motor cortex between control and ALS (Figure 8c; *p* = 0.65).

## 3. Discussion

Whilst extensive research into the pathogenesis of ALS has implicated many neuronal pathways, the precise drivers of disease onset and progression still remain unclear. The interrogation of oligodendrocytes and myelin changes has begun to receive more attention, but still remains relatively superficial. Here, we provide a comprehensive characterisation of the white matter of the motor cortex, to understand how myelin composition is altered in ALS. Indeed, we found altered lipid metabolism in the motor cortex white matter, as well as the altered gene expression of relevant lipid metabolising enzymes and transporters, which suggests the lipid composition of myelin may be different in ALS. With respect to myelin protein composition, we found no change in the levels of key myelin proteins. Thus, we report a shift in myelin composition, although there was no change in the total amount of myelin, which is driven by altered lipid metabolism. Given that changes in the myelin structure alter neuronal function, these findings highlight the important need to consider the contribution of oligodendrocytes to ALS pathogenesis.

We found a significant increase in cholesterol levels in ALS motor cortex white matter compared to controls. The myelin sheath is the only biological structure that has such a heavily skewed proportion of lipids to protein, compared to an approximate equivalence in most biological membranes [21]. Further, myelin has the highest proportion of cholesterol compared to other biological membranes [22]. Cholesterol is essential for myelin synthesis and stability [23], and myelin cholesterol arises from de novo synthesis in oligodendrocytes as well as from neighbouring astrocytes. Thus, our detection of increased cholesterol levels in the motor cortex white matter likely reflects a change in myelin cholesterol levels. This has important ramifications in the context of ALS given that tight regulation of cholesterol levels is imperative to normal central nervous system functioning. In fact, dysregulation of cholesterol levels is associated with neurodegenerative diseases, including Alzheimer’s disease, Huntington’s disease and Parkinson’s disease [24]. Our data also suggest that ALS patients lack the ability to tightly regulate cholesterol metabolism and this is reflected in the correlation analyses between cholesterol levels and its associated enzymes. Future studies would also benefit from characterising the functional capacity of these associated enzymes. Finally, it has been reported that ALS patients are prone to cholesterol droplet build-up [25] and whilst this has most commonly been attributed to astrocytes to date, cholesterol droplets have also been identified in oligodendrocytes [26]. Increased cholesterol has previously been reported in ALS (in human CSF [27], human spinal cord ventral horn [28] and the spinal cord of SOD1 mutant mice [17,28]); our data suggest that oligodendrocytes and myelin may also contribute to altered cholesterol levels.

In addition to the increased cholesterol, our lipidomic analyses also highlighted reductions in ceramide, glycerolipid, phospholipids and sphingomyelin; all of which are integral lipid components of myelin [23]. We acknowledge that we cannot conclude that our findings are entirely representative of myelin given that there are no myelin-specific lipids, but white matter lipid signatures are predicted to reflect myelin lipid content and this has been demonstrated in the neurodegenerative field previously [29]. What we can definitively conclude is that lipid metabolism is altered in the motor cortex white matter and we argue this is driven by changes to oligodendrocyte metabolism and myelin composition. Whilst we are the first to characterise lipid metabolism in the white matter in human ALS tissue, previous studies in ALS rodent models and post-mortem tissue have identified dysregulated lipid metabolism in the grey matter and also identified lipids other than cholesterol that are dysregulated. Henriques and colleagues assayed lipid expression in the whole lumbar spinal cord of symptomatic SOD1^G86R^ mouse (therefore white and grey matter) and found altered sphingomyelin, ceramide and phosphatidylcholine levels, which are all key constituents of myelin [30]. Similarly, in the SOD1^G93A^ mouse, large lipidomic changes were noted in end-stage disease in the spinal cord, whereas lipidomic changes in the motor cortex were mostly related to age [17]. Our control samples are age-matched to our ALS cases, thereby excluding this as an impacting variable. This also strengthens the association between the lipid changes being oligodendrocyte-driven, as opposed to being secondary changes to neuronal loss, given the low neuronal loss in the motor cortex relative to tissue volume. Of greater relevance to our data, the investigation of lipid metabolism has also begun in TDP-43 mouse models; mice over-expressing TDP-43 exhibit increased fat accumulation, as well as adipocyte hypertrophy [31] and mice harbouring the pathogenic Q331K point mutation in the human *TDP-43* transgene are also overweight [32]. Thus, lipid metabolism dysregulation in ALS is not a new concept; however, its link with oligodendrocyte function is underappreciated. There is one study that aimed to characterise myelin composition using the SOD1^G93A^ rat by using a gradient to isolate myelin from the lumbar spinal cord; despite the relatively low resolution of this technique, they also showed decreased cerebrosides and phospholipids, but also showed decreased cholesterol [33]. A study assaying lipid composition of the motor cortex in patients with multiple system atrophy (MSA), an oligodendrocyte-driven disease, also identified decreased sphingomyelins, sulfatides and galactosylceramides, which they attributed to changes in myelin composition [29]. With respect to oligodendrocytes, we know that monocarboxylate transporter 1 (MCT1; an oligodendrocyte specific transporter) not only shuttles lactate, but also ketone bodies. Reduced MCT1 results in neurons being at a heightened risk to age-related disorders including ALS [34] and it has been shown that MCT1 is downregulated in ALS [4,13] and oligodendrocytes have a reduced capacity to traffic lactate through MCT1 in ALS [9]. Therefore, reduced MCT1 expression in ALS could also potentially contribute to altered lipid metabolism. Thus, oligodendrocytes with their lipid dense myelin sheath likely play a pivotal role in dysregulated lipid homeostasis in ALS.

Despite the changes observed in lipid levels, we did not detect any change in the expression of key myelin proteins in the white matter of the motor cortex in ALS cases. The analyses of the oligodendrocyte contribution to ALS have, to a large degree, been unclear and conflicting. The expression levels of myelin proteins have been shown to be increased [4], decreased [13] and not changed [10,19] with expression levels also varying depending on the region assessed. Thus, it cannot be ruled out that neuronal loss is contributing to myelin loss, as opposed to the myelin change being oligodendrocyte-driven and therefore a specific phenotype of ALS. Indeed, we assayed the motor cortex from cases mostly diagnosed with limb-onset ALS to more accurately elucidate the contribution of the oligodendrocyte to the changes in myelin, as opposed to the changes being secondary to Wallerian degeneration. Whilst we did not find any statistical differences in myelin protein expression, there was a trend for reduced PLP and MOG. MOG had not previously been characterised in the context of ALS and we know it is essential for the integrity of the myelin sheath. Decreased PLP has been identified in mice that have the *TDP-43* gene deleted from mature oligodendrocytes [8], albeit these mice also exhibited decreased MBP, which we did not observe. PLP is known to be trafficked to the myelin sheath along lipid rafts and could therefore possibly tie in to the lipid dysregulation we also observed; indeed, this pattern has been identified previously in MSA [35]. Mutations in the *PLP* gene have also been associated with type 2 spastic paraplegia, which is classified as a type of motor neuron disease [36]. We utilised SCoRe imaging to determine if the amount of myelin was altered in ALS cases compared to controls and found no change. As stated, the majority of myelin assessment in an ALS context is limited to Western blotting for individual proteins, which comprise only a portion of the 30% protein component of myelin. Our assessment of myelin density supports previous g-ratio analyses [10] suggesting no change to the amount of myelin in the white matter of the motor cortex. These findings are in contrast to what has been shown previously in the spinal cord [12,13,19], further supporting the contention that myelin loss in the spinal cord is likely secondary to motor neuron loss as opposed to it being oligodendrocyte-driven. It is interesting that we show no change in the amount of myelin despite finding significant lipid changes which also suggests that oligodendrocytes have the capacity to maintain myelin levels despite the shift in its composition. The impact of this changed composition on myelin function, transporter/receptor localisation and myelin integrity remains unexplored and certainly warrants further investigation.

Thus, we are the first to comprehensively characterise the white matter of the motor cortex in ALS and definitively show altered lipid levels, suggesting dysregulated lipid homeostasis. We also assayed the expression levels of key myelin proteins and found there was no difference in ALS cases. Thus, we conclude that myelin composition is likely altered in ALS and it is driven by changes in the lipid metabolism, and not changes in the protein expression. Therefore, if oligodendrocytes are dysfunctional in ALS, then it seems likely that they may contribute to neuronal dysfunction and death in ALS.

## 4. Materials and Methods

### 4.1. Tissue Preparation

Human post-mortem tissue was kindly provided by the Victorian Brain Brank; use of this tissue was approved by the Medicine and Dentistry Human Ethics Sub-Committee of the Human Research Ethics Committee at the University of Melbourne; ethics #1852824. The clinical data for each patient sample can be found in Table 1; briefly, we included sporadic ALS cases (sALS; n = 8; used for all analyses) and familial ALS cases (fALS; n = 6; used for qRT-PCR and Western blotting analyses). The controls (n = 8) were age- and sex-matched to the sALS cases. The Brodmann Area 4 (BA4) region of the brain was provided by the Victorian Brain Bank and our analyses focused solely on the white matter.

### 4.2. Targeted Lipidomic Analyses of the Motor Cortex White Matter

White matter from the motor cortex was dissected (20–30 mg) from frozen BA4 brain samples from sALS (n = 8) and control (n = 8) cases. For lipid extraction, the tissue was chilled in 600 uL methanol:chloroform (9:1) containing 10 mg/L of each internal standard. The internal standards were PC19:0/19:0 (part 850367), PE-d31 (part 860374), PG17:0/17:0 (part 830456) and TG-d5 19:0/12:0/19:0 (part 8609040) from Avanti Polar Lipids, Alabama, USA. Samples were homogenised using a cryomill at 6800 RPM for 3 × 45 s cycles. Then, 400 uL of the homogenate was transferred into fresh LoBind Eppendorf tubes and 680 uL chloroform was added to each tube to bring the ratio of chloroform:methanol to 2:1. Samples were vortexed and then mixed at 950 rpm for 30 min at 20 °C with a Thermomixer (Eppendorf South Pacific Pty Ltd., Macquarie Park, Australia). Samples were centrifuged at 15,000 rpm (Beckman Coulter Microfuge^®^ 22R Refrigerated Microcentrifuge, Beckman Coulter Australia Pty Ltd., Sydney, Australia) for 10 min and the supernatant was transferred to fresh LoBind Eppendorf tubes. Samples were completely dried in a vacuum concentrator with the temperature maintained at 30–35 °C (Christ^®^ RVC 2–33, Martin Christ Gefriertrocknungsanlagen GmbH, Osterode am Harz, Germany). The samples were reconstituted with methanol:water-saturated butanol (100 µL, 1:9, *v*/*v*). Pooled biological quality control samples (PBQC) were prepared by pooling aliquots of the extracts from each sample and were run after every four samples. Extracted lipids were processed and detected by Metabolomics Australia (Bio21 Institute, Melbourne, VIC, Australia) as previously described [37,38] using an Agilent 1290 liquid chromatography (LC) system and Triple Quadrupole 6490 mass spectrometer (MS, Agilent Technologies Australia, Mulgrave, Australia). For LCMS analysis, nonpolar (lipid) extracts were analyzed by LCMS in positive ionisation mode to obtain the most comprehensive coverage with dynamic scheduled multiple reaction monitoring. The MS parameters and MRM transitions of each lipid class, subclass and individual lipid species have been previously described [37,38]. Data processing was performed using Agilent’s Mass Hunter Quantitative Analysis (QQQ) software (Agilent Technologies Australia). The final data matrix was then log transformed and normalised to the median for statistical analyses. For graphing, data were normalised to the median only. Regression analyses showed no correlation between PMI and the differences observed in lipid expression levels (data not shown). Lipid species were named according to the LIPID MAPS nomenclature described previously [39].

### 4.3. Quantitative RT-PCR for Gene Expression of Metabolic Enzymes

White matter from the motor cortex was dissected (20–30 mg) from frozen BA4 brain samples from sALS (n = 8), fALS (n = 6) and control (n = 8) cases; sALS and fALS cases were combined for graphical and statistical presentation (ALS). Tissue was homogenised in QIAzol Lysis Reagent (Qiagen, Chadstone, VIC, Australia, 79306) in a TissueLyser LT Adaptor and RNA extracted using the RNeasy Mini Kit (Qiagen, 74106) with in-column DNase digestion. Briefly, 1 mL QIAzol lysis buffer was added to the frozen brain sample in each Eppendorf and lysed for 2–5 min at 50 Hz in the TissueLyser until no debris remained. To each tube, 200 µL of chloroform was added and each tube was shaken vigorously. Tubes were centrifuged at 12,000× *g* for 15 min at 4 °C and the upper aqueous phase was transferred to a new Eppendorf, to which 70% ethanol was added and the entire solution was transferred to a RNeasy Mini spin column. The remainder of the process was as per the manufacturer’s instructions (Qiagen). RNA (400 ng) was transcribed into cDNA using iScript Reverse Transcription Supermix (Bio-Rad, Chadstone, VIC, Australia, 1708841). The concentration of cDNA used per qRT-PCR reaction was gene dependent (2.5 ng per well for *ABCA1*, *ApoE*, *CEPT1*, *CYP46A1*, *DEGS1*, *DGAT1*, *GALC*, *HMGCR*, *HMGCS1*, *LPIN1*, *SGMS1*, *SMPD1* and 20 ng per well for *ASAH1, CERS1, SCAP, UTG8)* and SsoAdvanced Universal SYBR Green Supermix (Bio-Rad, 1725274) was used. Primer sequences can be found in Table 2; annealing temperatures were determined for optimal amplification and single peak melt curves. All samples were run in triplicate with a no cDNA template control and a no reverse transcriptase control. Data were analysed using the delta delta CT (∆∆CT) method and were normalised to the square root of two housekeeping genes (18S and ACTB). ALS samples were presented as a fold change to the average of all controls.

### 4.4. Correlation of Lipid Expression with Enzymatic Gene Expression and Disease Duration

To determine the correlation between lipid species expression and the gene expression of the enzymes responsible for the respective lipid’s metabolism, an average lipid level was calculated for all species within that lipid family per sample and generated as a fold change to the average of all controls to match data handling of the qRT-PCR output. The average lipid level was then correlated to its respective enzyme expression and a line of best fit drawn and the r-value was used for statistical comparison between ALS and control. Cholesterol ester level was also correlated to disease duration for each patient and the r^2^-value was analysed statistically.

### 4.5. Western Blotting for Myelin Proteins

White matter from the motor cortex was dissected (20–30 mg) from frozen BA4 brain samples from sALS (n = 8), fALS (n = 6) and control (n = 8) cases; sALS and fALS cases were combined for graphical and statistical presentation (ALS). Tissue was homogenised in RIPA buffer (50 mM Tris-Cl, pH 7.4, 150 mM NaCl, 1% Triton X-100, 0.1% SDS, 1% sodium deoxycholate) plus phosphatase inhibitors (50 mM NaF, 10 mM Na_3_VO_4_) and protease inhibitors (Sigma, Bayswater, WA, Australia, P8340) using sonification. The soluble protein fraction was analysed using Western blotting. The concentration of protein used for each blot was determined for each antibody and are listed below. Protein was denatured in Laemmli buffer with 10% beta-mercaptoethanol at 100 °C for 5 min. For probing with PLP, protein was incubated in Laemmli buffer with 7M urea, 2M thiourea and 4% beta-mercaptethanol at 37 °C for 15–30 min. Samples were separated by electrophoresis using 4–20% Mini-Protean TGX Stain-Free gel (Bio-Rad) and two gels were required per antibody so that control and ALS samples were randomised across gels to reduce gel bias. The protein was transferred onto an Immun-Blot PVDF membrane (0.2 µm pore size; Bio-Rad) using the Trans-Blot Turbo Transfer System (Bio-Rad). Membranes were blocked in 5% skim milk powder in Tris-buffered saline with 0.15% Tween-20 (TBST) and 0.02% NaN_3_ before incubation with primary antibodies overnight at 4 °C in 3% BSA in TBST with 0.02% NaN_3_ (MBP Ab7349 1:500 1 µg protein; CNPase AMAB91072 1:1000 10 µg protein; MOG MAB96457 1:1000 10 µg protein; MAG MAB9043 1:1000 10µg protein; PLP/DM20 MAB388 1:500 20µg protein). Membranes were incubated in the relevant secondary antibodies (LiCor BioSciences, Lincoln, NE, USA) for 30–45 min at RT. A housekeeping protein (β-actin Bio-Rad #12004163 1:5000) was run on all blots as a loading control. Protein was visualised on a ChemiDoc Imager (Bio-Rad). Analysis was done using the gel plugin on the ImageJ software, wherein the intensity of each protein band was measured and normalised to β-actin for each sample. Due to two protein bands produced by PLP/DM20, quantification was performed manually by taking the integrated intensity of each protein band, subtracting background noise and then normalising it to the integrated density of β-actin. Protein expression was calculated as a fold change to the average of all controls.

### 4.6. SCoRe Imaging

Cryosections of fresh frozen motor cortex (50µm) from ALS (n = 8 sALS) and control (n = 8) were fixed in 4% paraformaldehyde for 60 min at RT. Sections were incubated with Hoechst 33342 (1:1000, Invitrogen, Waltham, MA, USA, H3570) to aid with distinguishing grey from white matter and mounted (DAKO Mounting Medium, Agilent Technologies, Mulgrave, VIC, Australia, CS703). All images were captured on a Zeiss LSM 880 confocal microscope using an optimised imaging protocol [40] using an oil immersion 40× objective; three images of the white matter were taken per tissue section between 5–20 µm below the coverslip. To perform quantitative analysis, the three imaged channels were overlaid into a composite image and analysed by positive pixel identification by using a minimum threshold cutoff in the Fiji Image J software. Areas of damage and also blood vessels exceeding 10 µm in diameter were removed from the areal analyses of each image prior to pixel measurement. The ratio of positive pixels was expressed as a proportion of area and then the ALS cases were each calculated as a fold change from the average of all controls.

### 4.7. Fluorescent Immunocytochemistry for OLIG2

Cryosections of fresh frozen motor cortex (16 µm) were fixed in 4% paraformaldehyde for 30 min at RT. Antigen retrieval was carried out using 1% sodium lauryl sulfate (SDS) for 5 min at RT. Sections were blocked with 10% normal donkey serum in 0.3% Triton X-100 in PBS for 1 h at RT before overnight incubation with the primary antibody at 4 °C (Olig2, 1:100, Millipore, Bayswater, WA, Australia, MAB9610). Sections were then incubated in secondary antibody (AlexaFluor-488, 1:400, Jackson Immuno, West Grove, PA, USA, 711545152) for 2 h at RT. Sections were incubated with Hoechst 33342 (1:1000, Invitrogen, H3570) for 10 min and mounted (DAKO Mounting Medium, Agilent Technologies, CS703). The white matter was visualised using a Zeiss AxioObserver ZI and ten images were taken per slide using a 20× objective. The percentage of OLIG2-positive cells to total density of Hoescht+ nuclei in the white matter for each sample was determined.

### 4.8. Statistical Analyses

For the individual lipid species, qRT-PCR, Western blot and immunocytochemistry, data were analysed using an unpaired *t*-test. The heatmaps, PCA plots, volcano plot, PLS-DA plot and VIP score plot were performed using MetaboAnalyst software. For correlation analyses between lipid expression and enzyme expression, linear regression analyses were conducted comparing the r-values for control versus ALS. For correlation analyses between lipid expression and disease duration, a linear regression analysis was conducted to ascertain the r^2^- and *p*-value. Data are presented as mean ± SEM. * denotes *p* < 0.05, ** denotes *p* < 0.01, *** denotes *p* < 0.001.

## Figures and Tables

**Table 1:** Clinical data of human post-mortem tissue samples used.

Case	Diagnosis	Age *	Sex	PMI #	Region	Onset	Duration *
1	Control	73.6	M	49	BA4	/	/
2	Control	52.1	M	33	BA4	/	/
3	Control	63.9	M	68	BA4	/	/
4	Control	75.9	M	50	BA4	/	/
5	Control	77.5	M	28	BA4	/	/
6	Control	57.6	M	20.5	BA4	/	/
7	Control	73.5	M	22	BA4	/	/
8	Control	79.6	M	31.5	BA4	/	/
9	fALS	43.8	M	77	BA4	Limb-lower	1.6
10	fALS	58.4	M	24.5	BA4	Limb-upper	2.5
11	fALS	64.3	F	39.5	BA4	Limb-upper (left)	2.5
12	fALS	69.3	F	45	BA4	Limb-upper & lower (right)	3.0
13	fALS	72.8	M	21.5	BA4	Limb-lower (left)	3.0
14	sALS	41.6	M	42	BA4	Limb-lower	2.3
15	sALS	53	M	29	BA4	Limb-lower (right)	6.0
16	sALS	64.8	M	45	BA4	Limb-lower	6.5
17	sALS	75.7	M	26	BA4	Limb-upper & lower	2.8
18	sALS	78.1	M	13.5	BA4	Bulbar	3.5
19	sALS	66.9	M	21	BA4	Limb-lower (left)	1.3
20	sALS	57.6	M	21.5	BA4	Limb-upper & lower (left)	1.1
21	sALS	66.1	M	32.5	BA4	Limb-lower	5.75
22	fALS	41.9	F	30.5	BA4	Limb-upper & lower	0.1 (7 weeks)

* measured in years. # measured in hours. sALS = sporadic ALS; fALS = familial ALS.

**Table 2:** Primers used for the qRT-PCR reactions with their optimised annealing temperature.

Gene Name	Forward Primer	Reverse Primer	Anneal Temp (°C)
*ASAH1*	5′-GCACAAGTTATGAAGGAAGCCAAG	5′-TCCAATGATTCCTTTCTGTCTCG	57
*CERS1*	5′-CTGCTCCAGGGAAGCTTCTA	5′-GGAGGAGACGATGAGGATGA	59
*DEGS1*	5′-GCAAAGCAATGTGGAATCGC	5′-ACCAGCCCTCAAAATCGGTA	57
*SGMS1*	5′-TGAGCCTCTGGAGCATTTCA	5′-CCGTTCTTGTGTGCTTCCAA	61
*SMPD1*	5′-CAGACTCGTCAGGACCAACT	5′-GGGGAGGGAAGCTATTGACA	64
*UGT8*	5′-ATGGGTAAATGGTGCTAATG	5′-TCTGGTCATAGTATCATAATGG	63
*GALC*	5′-GGCTCATTATCCTGGAACCC	5′-GCCTGCACCCATGTCACTAT	57
*HMGCS1*	5′-TGTACACATCTTCAGTATATGGTTCCC	5′-AAGAAAACACTCCAATTCTCTTCCCT	57
*HMGCR*	5′-TGCTCGTGGAATGGCAATTT	5′-ACCAACTCCAATCACAAGACA	63
*SCAP*	5′-TGGGGATGTCACCTCCCTTAC	5′-TGCTGAATGGAGTAGAACTTGATGC	59
*ABCA1*	5′-TGCAAGGCTACCAGTTACATT	5′-TTAGTGTTCTCAGGATTGGCT	59
*ApoE*	5′-TGGACGAGGTGAAGGAGCAG	5′-CTCGAACCAGCTCTTGAGGC	57
*CYP46A1*	5′-CAGGGAAGAGGAAGCAGCTC	5′-ATCTGTGTGAGGATGTCGGC	60
*CEPT1*	5′-GCGGGATCCATGAGTGGGCATCGATCAACA	5′-GCGGTCGACTTAATGATTAGAATGAGCTGT	63
*LPIN1*	5′-TGAAAAGGGGCTCTGTGGAC	5′-ACTACAGAGCTGCTTGACGG	59
*DGAT1*	5′-TGCAGGATTCTTTATTCAGCTCT	5′-GCATCACCACACACCAGTTC	57
*MBP*	5′-CTTCTTTGGCGGTGACAGG	5′-CGGGGTTTTCATCTTGGGTC	57
*ACTB*	5′-GTTACAGGAACTCCCTTGCCATCC	5′-CACCTCCCCTGTGTGGACTTGGG	57
*18S*	5′-GTAACCCGTTGAACCCCATT	5′-CCATCCAATCGGTAGTAGCG	59
*ASAH1*	5′-GCACAAGTTATGAAGGAAGCCAAG	5′-TCCAATGATTCCTTTCTGTCTCG	57
*CERS1*	5′-CTGCTCCAGGGAAGCTTCTA	5′-GGAGGAGACGATGAGGATGA	59
*DEGS1*	5′-GCAAAGCAATGTGGAATCGC	5′-ACCAGCCCTCAAAATCGGTA	57

## Data Availability

Data are available upon request. The data are not publicly available currently as they are being mined in conjunction with other studies.

## Supplementary Materials

The following are available online at https://www.mdpi.com/article/10.3390/metabo12060554/s1, Figure S1: Targeted lipidomic analyses of motor cortex white matter in ALS versus control; Figure S2: A schematic diagram demonstrating the relationship between identified lipids and their asso-ciated enzymes; Figure S3: There is less regulation of cholesterol metabolism in ALS; Figure S4: There is significant difference in the expression of key lipid metabolizing enzymes relative to their respective lipid species in ALS; Figure S5: There is no difference in the density of OLIG2+ oligodendrocytes in the motor cortex white matter in ALS compared to control.

## References

[1] O’Reilly E.J., Wang H., Weisskopf M.G., Fitzgerald K., Falcone G., McCullough M.L., Thun M., Park Y., Kolonel L.N., Ascherio A. (2013). Premorbid body mass index and risk of amyotrophic lateral sclerosis. Amyotroph. Lateral Scler. Front. Degener..

[2] Wills A.-M., Hubbard J., Macklin E., Glass J., Tandan R., Simpson E.P., Brooks B., Gelinas D., Mitsumoto H., Mozaffar T. (2014). Hypercaloric enteral nutrition in patients with amyotrophic lateral sclerosis: A randomised, double-blind, placebo-controlled phase 2 trial. Lancet.

[3] Fünfschilling U., Supplie L.M., Mahad D., Boretius S., Saab A.S., Edgar J., Brinkmann B.G., Kassmann C.M., Tzvetanova I.D., Möbius W. (2012). Glycolytic oligodendrocytes maintain myelin and long-term axonal integrity. Nature.

[4] Lee Y., Morrison B.M., Li Y., Lengacher S., Farah M.H., Hoffman P.N., Liu Y., Tsingalia A., Jin L., Zhang P.-W. (2012). Oligodendroglia metabolically support axons and contribute to neurodegeneration. Nature.

[5] Rinholm J.E., Bergersen L.H. (2012). Neuroscience: The wrap that feeds neurons. Nature.

[6] Saab A.S., Tzvetanova I.D., Nave K.-A. (2013). The role of myelin and oligodendrocytes in axonal energy metabolism. Curr. Opin. Neurobiol..

[7] Neumann M., Kwong L.K., Truax A.C., Vanmassenhove B., Kretzschmar H.A., Van Deerlin V.M., Clark C.M., Grossman M., Miller B.L., Trojanowski J.Q. (2007). TDP-43-Positive White Matter Pathology in Frontotemporal Lobar Degeneration with Ubiquitin-Positive Inclusions. J. Neuropathol. Exp. Neurol..

[8] Wang J., Ho W.Y., Lim K., Feng J., Tucker-Kellogg G., Nave K.-A., Ling S.-C. (2018). Cell-autonomous requirement of TDP-43, an ALS/FTD signature protein, for oligodendrocyte survival and myelination. Proc. Natl. Acad. Sci. USA.

[9] Ferraiuolo L., Meyer K., Sherwood T.W., Vick J., Likhite S., Frakes A., Miranda C.J., Braun L., Heath P.R., Pineda R. (2016). Oligodendrocytes contribute to motor neuron death in ALS via SOD1-dependent mechanism. Proc. Natl. Acad. Sci. USA.

[10] Barton S.K., Gregory J.M., Selvaraj B.T., McDade K., Henstridge C.M., Spires-Jones T.L., James O.G., Mehta A.R., Story D., Burr K. (2021). Dysregulation in Subcellular Localization of Myelin Basic Protein mRNA Does Not Result in Altered Myelination in Amyotrophic Lateral Sclerosis. Front. Neurosci..

[11] Barton S.K., Magnani D., James O.G., Livesey M.R., Selvaraj B.T., James O.T., Perkins E.M., Gregory J.M., Cleary E., Ausems C.R.M. (2021). Transactive response DNA-binding protein-43 proteinopathy in oligodendrocytes revealed using an induced pluripotent stem cell model. Brain Commun..

[12] Pons A.L., Higginbottom A., Cooper-Knock J., Alrafiah A., Alofi E., Kirby J., Shaw P., Wood J.D., Highley J.R. (2020). Oligodendrocyte pathology exceeds axonal pathology in white matter in human amyotrophic lateral sclerosis. J. Pathol..

[13] Kang S.H., Li Y., Fukaya M., Lorenzini I., Cleveland D., Ostrow L., Rothstein J.D., Bergles D.E. (2013). Degeneration and impaired regeneration of gray matter oligodendrocytes in amyotrophic lateral sclerosis. Nat. Neurosci..

[14] Thornton M.A., Hughes E.G. (2020). Neuron-oligodendroglia interactions: Activity-dependent regulation of cellular signaling. Neurosci. Lett..

[15] Chen T.-J., Kula B., Nagy B., Barzan R., Gall A., Ehrlich I., Kukley M. (2018). In Vivo Regulation of Oligodendrocyte Precursor Cell Proliferation and Differentiation by the AMPA-Receptor Subunit GluA2. Cell Rep..

[16] Morell P., Quarles R.H. (1999). Characteristic Composition of Myelin. Basic Neurochemistry: Molecular, Cellular and Medical Aspects.

[17] Filho A.D.B.C., Pinto I.F.D., Dantas L.S., Xavier A.M., Inague A., Faria R.L., Medeiros M.H.G., Glezer I., Yoshinaga M.Y., Miyamoto S. (2019). Alterations in lipid metabolism of spinal cord linked to amyotrophic lateral sclerosis. Sci. Rep..

[18] Blasco H., Veyrat-Durebex C., Bocca C., Patin F., Vourc’h P., Nzoughet J.K., Lenaers G., Andres C., Simard G., Corcia P. (2017). Lipidomics Reveals Cerebrospinal-Fluid Signatures of ALS. Sci. Rep..

[19] Philips T., Bento-Abreu A., Nonneman A., Haeck W., Staats K., Geelen V., Hersmus N., Küsters B., Van Den Bosch L., Van Damme P. (2013). Oligodendrocyte dysfunction in the pathogenesis of amyotrophic lateral sclerosis. Brain.

[20] Chang K.-J., Agrawal I., Vainshtein A., Ho W.Y., Xin W., Tucker-Kellogg G., Susuki K., Peles E., Ling S.-C., Chan J.R. (2021). TDP-43 maximizes nerve conduction velocity by repressing a cryptic exon for paranodal junction assembly in Schwann cells. eLife.

[21] Williams K.A., Deber C.M., Klrschner O.A. (1993). The Structure and Function of Central Nervous System Myelin. Crit. Rev. Clin. Lab. Sci..

[22] O’Brien J.S. (1965). Stability of the Myelin Membrane. Science.

[23] Poitelon Y., Kopec A.M., Belin S. (2020). Myelin Fat Facts: An Overview of Lipids and Fatty Acid Metabolism. Cells.

[24] Zhang J., Liu Q. (2015). Cholesterol metabolism and homeostasis in the brain. Protein Cell.

[25] Pennetta G., Welte M. (2018). Emerging Links between Lipid Droplets and Motor Neuron Diseases. Dev. Cell.

[26] Smolič T., Zorec R., Vardjan N. (2021). Pathophysiology of Lipid Droplets in Neuroglia. Antioxidants.

[27] Abdel-Khalik J., Yutuc E., Crick P.J., Gustafsson J., Warner M., Roman G., Talbot K., Gray E., Griffiths W.J., Turner M.R. (2017). Defective cholesterol metabolism in amyotrophic lateral sclerosis. J. Lipid Res..

[28] Cutler R.G., Pedersen W.A., Camandola S., Rothstein J.D., Mattson M.P. (2002). Evidence that accumulation of ceramides and cholesterol esters mediates oxidative stress-induced death of motor neurons in amyotrophic lateral sclerosis. Ann. Neurol..

[29] Don A.S., Hsiao J.-H.T., Bleasel J.M., Couttas T.A., Halliday G.M., Kim W.S. (2014). Altered lipid levels provide evidence for myelin dysfunction in multiple system atrophy. Acta Neuropathol. Commun..

[30] Henriques A., Croixmarie V., Bouscary A., Mosbach A., Keime C., Boursier-Neyret C., Walter B., Spedding M., Loeffler J.-P. (2018). Sphingolipid Metabolism Is Dysregulated at Transcriptomic and Metabolic Levels in the Spinal Cord of an Animal Model of Amyotrophic Lateral Sclerosis. Front. Mol. Neurosci..

[31] Stallings N.R., Puttaparthi K., Dowling K.J., Luther C.M., Burns D.K., Davis K., Elliott J.L. (2013). TDP-43, an ALS Linked Protein, Regulates Fat Deposition and Glucose Homeostasis. PLoS ONE.

[32] Watkins J.A., Alix J.J.P., Shaw P.J., Mead R.J. (2021). Extensive phenotypic characterisation of a human TDP-43Q331K transgenic mouse model of amyotrophic lateral sclerosis (ALS). Sci. Rep..

[33] Niebroj-Dobosz I., Rafałowska J., Fidziańska A., Gadamski R., Grieb P. (2007). Myelin composition of spinal cord in a model of amyotrophic lateral sclerosis (ALS) in SOD1G93A transgenic rats. Folia Neuropathol..

[34] Raffaele S., Boccazzi M., Fumagalli M. (2021). Oligodendrocyte Dysfunction in Amyotrophic Lateral Sclerosis: Mechanisms and Therapeutic Perspectives. Cells.

[35] Wong J.H., Halliday G.M., Kim W.S. (2014). Exploring Myelin Dysfunction in Multiple System Atrophy. Exp. Neurobiol..

[36] Saugier-Veber P., Munnich A., Bonneau D., Rozet J.-M., Le Merrer M., Gil R., Boespflug-Tanguy O. (1994). X–linked spastic paraplegia and Pelizaeus–Merzbacher disease are allelic disorders at the proteolipid protein locus. Nat. Genet..

[37] Huynh K., Barlow C.K., Jayawardana K.S., Weir J.M., Mellett N.A., Cinel M., Magliano D., Shaw J.E., Drew B.G., Meikle P.J. (2019). High-Throughput Plasma Lipidomics: Detailed Mapping of the Associations with Cardiometabolic Risk Factors. Cell Chem. Biol..

[38] Huynh K., Pernes G., Mellett N.A., Meikle P.J., Murphy A.J., Lancaster G.I. (2018). Lipidomic Profiling of Murine Macrophages Treated with Fatty Acids of Varying Chain Length and Saturation Status. Metabolites.

[39] Liebisch G., Vizcaino J.A., Köfeler H., Trötzmüller M., Griffiths W., Schmitz G., Spener F., Wakelam M. (2013). Shorthand notation for lipid structures derived from mass spectrometry. J. Lipid Res..

[40] Gonsalvez D.G., Yoo S., Fletcher J., Wood R.J., Craig G.A., Murray S.S., Xiao J. (2019). Imaging and Quantification of Myelin Integrity After Injury with Spectral Confocal Reflectance Microscopy. Front. Mol. Neurosci..

