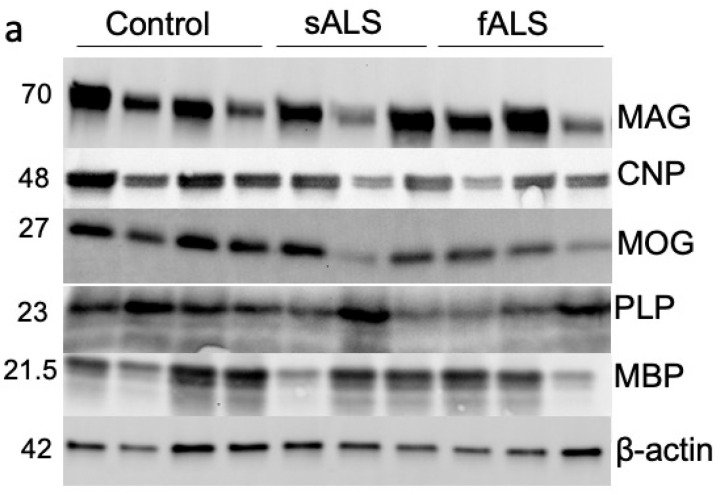# Correction: Sadler et al. Lipid Metabolism Is Dysregulated in the Motor Cortex White Matter in Amyotrophic Lateral Sclerosis. *Metabolites* 2022, *12*, 554

**DOI:** 10.3390/metabo15070486

**Published:** 2025-07-18

**Authors:** Gemma L. Sadler, Katherine N. Lewis, Vinod K. Narayana, David P. De Souza, Joel Mason, Catriona McLean, David G. Gonsalvez, Bradley J. Turner, Samantha K. Barton

**Affiliations:** 1 Florey Institute of Neuroscience and Mental Health, Melbourne 3052, Australia; gsadler1@student.unimelb.edu.au (G.L.S.); klewis3@student.unimelb.edu.au (K.N.L.); joel.mason@florey.edu.au (J.M.); bradley.turner@florey.edu.au (B.J.T.); 2 Metabolomics Australia, Bio21 Institute, University of Melbourne, Melbourne 3052, Australia; vinod.narayana@unimelb.edu.au (V.K.N.); desouzad@unimelb.edu.au (D.P.D.S.); 3 Victorian Brain Bank, Florey Institute of Neuroscience and Mental Health, Melbourne 3052, Australia; catriona.mclean@monash.edu; 4 Department of Anatomy and Developmental Biology, Monash University, Melbourne 3168, Australia; david.gonsalvez@monash.edu.au

## Error in Figure

In the original publication [1], there was a mistake in Figure 7a as published. The representative blots for the **MAG** and **CNP** proteins included an extra band. The corrected Figure 7a appears below. The authors state that the scientific conclusions are unaffected. This correction was approved by the Academic Editor. The original publication has also been updated.