# Supplementary material for: Lipid Metabolism Is Dysregulated in the Motor Cortex White Matter in Amyotrophic Lateral Sclerosis
Source: Metabolites. 2022 Jun 17;12(6):554. doi: 10.3390/metabo12060554 (PMC9230865; doi:10.3390/metabo12060554)
Supplement: Supplementary file 1 [file metabolites-12-00554-s001.zip › metabolites-1743515-SI.pdf]

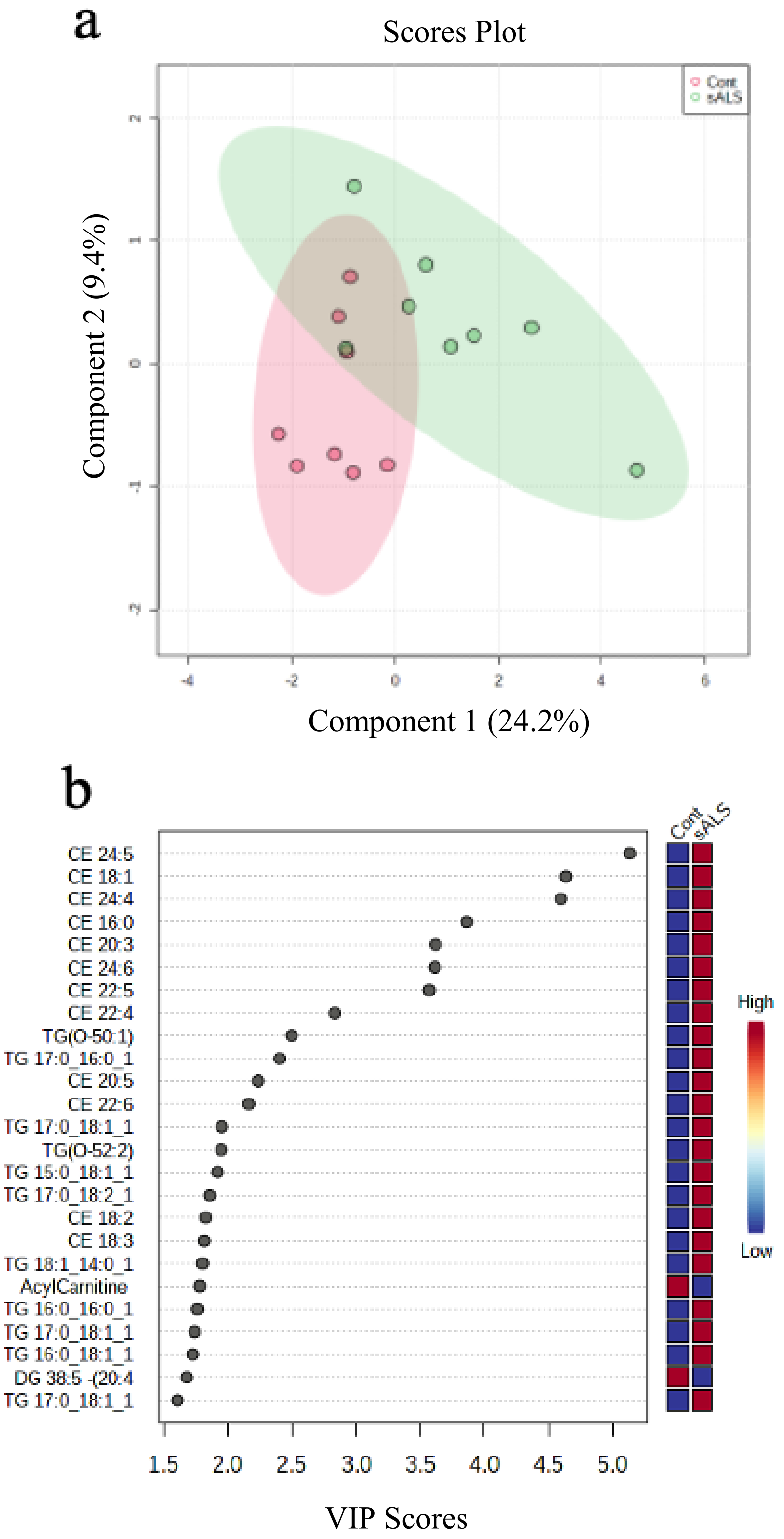

Supplementary Figure S1. Targeted lipidomic analyses of motor cortex white matter in ALS versus control (a) a PLS-DA plot depicting separation of ALS samples (sALS; n=8) from age- and sex-matched controls (n=8); (b) a VIP plot depicting the top 25 lipid species identified by the PLS-DA that differ between ALS and control

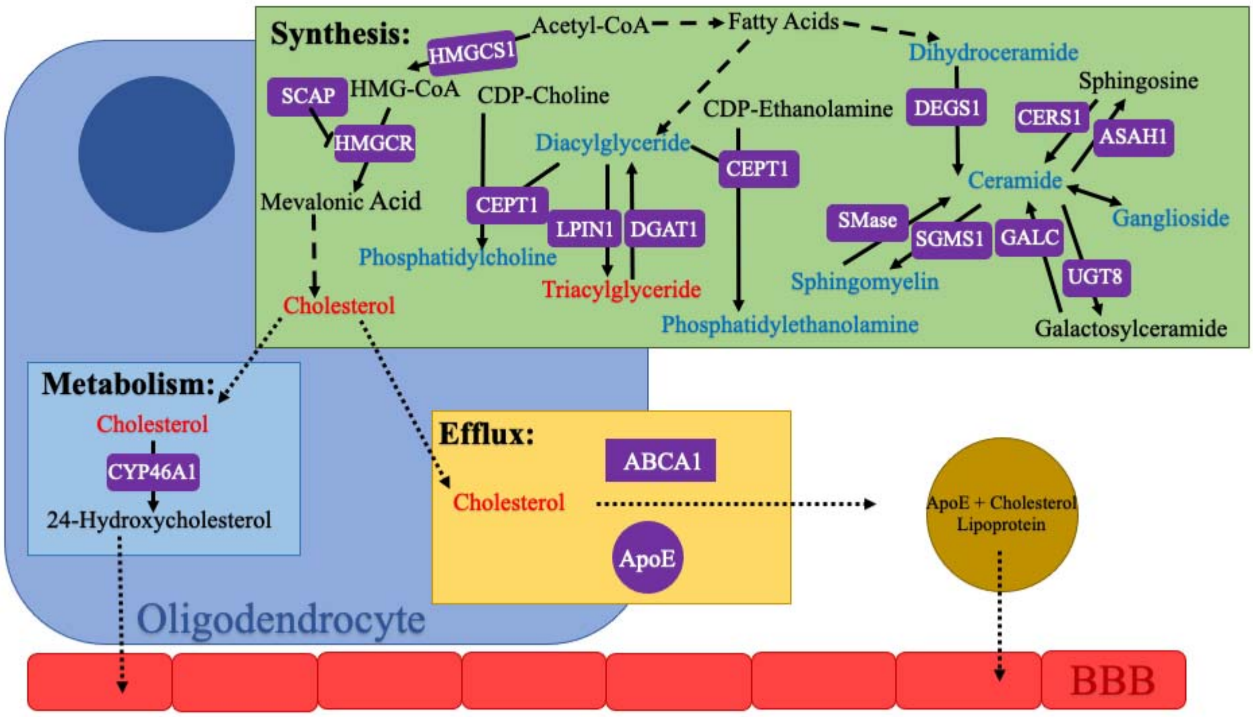

Supplementary Figure S2. A schematic diagram demonstrating the relationship between identified lipids and their associated enzymes. Lipid species identified as increased in ALS are in red and those that are decreased are identified in blue.

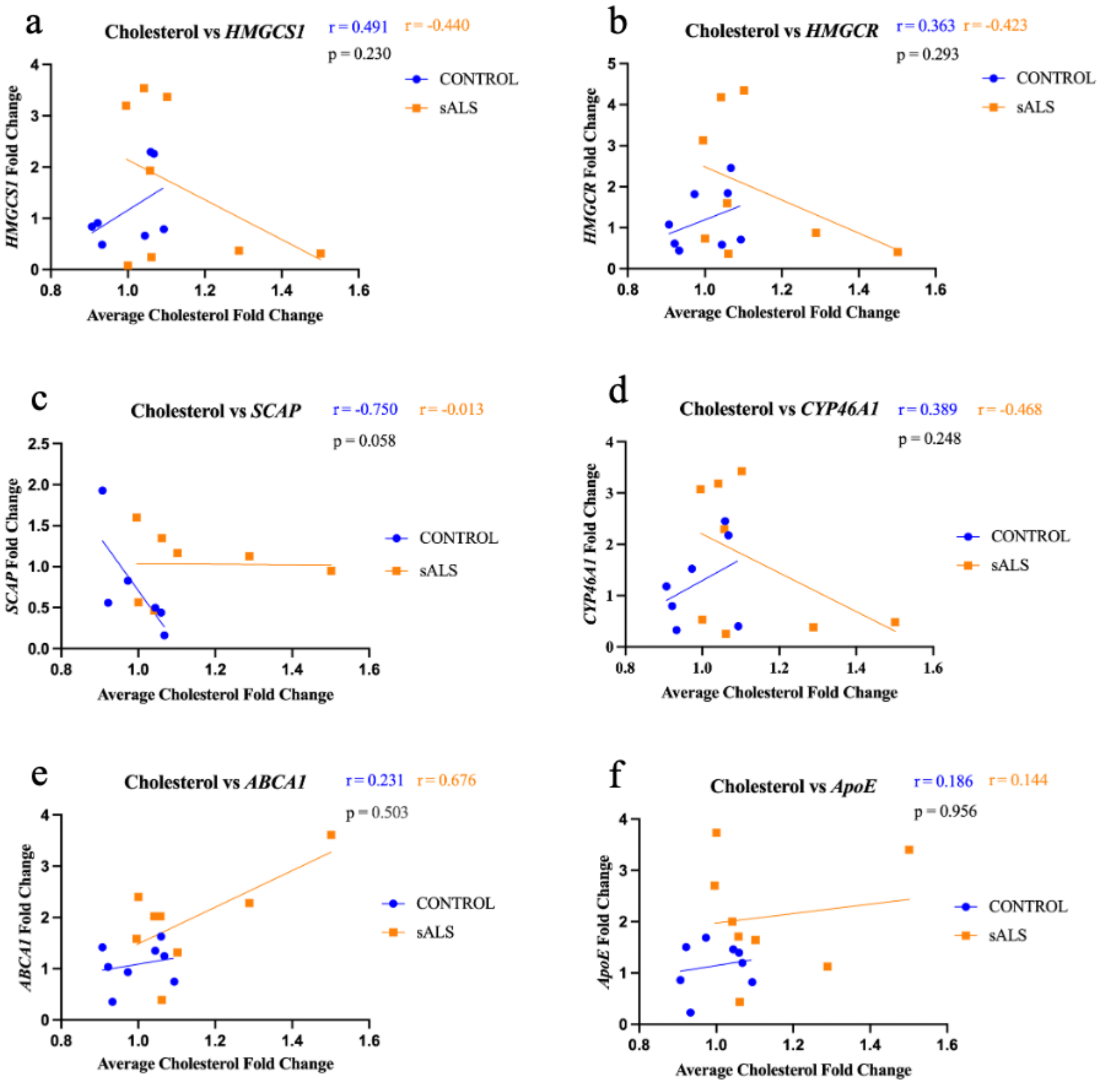

Supplementary Figure S3. There is less regulation of cholesterol metabolism in ALS. The correlation between cholesterol levels (expressed as a fold change of the average of all controls) and the gene expression of the enzymes (a) hydroxymethylglutaryl-CoA synthase (*HMGCS1*); (b) HMG-CoA reductase (*HMGCR*); (c) SREBP cleavage-activating protein (*SCAP*); (d) cholesterol 24-hydroxylase (*CYP46A1*); (e) ATP-binding cassette transporter A1 (*ABCA1*) and (f) Apolipoprotein E (*ApoE*) in ALS (sALS n=8; orange) and control (n=8; blue).

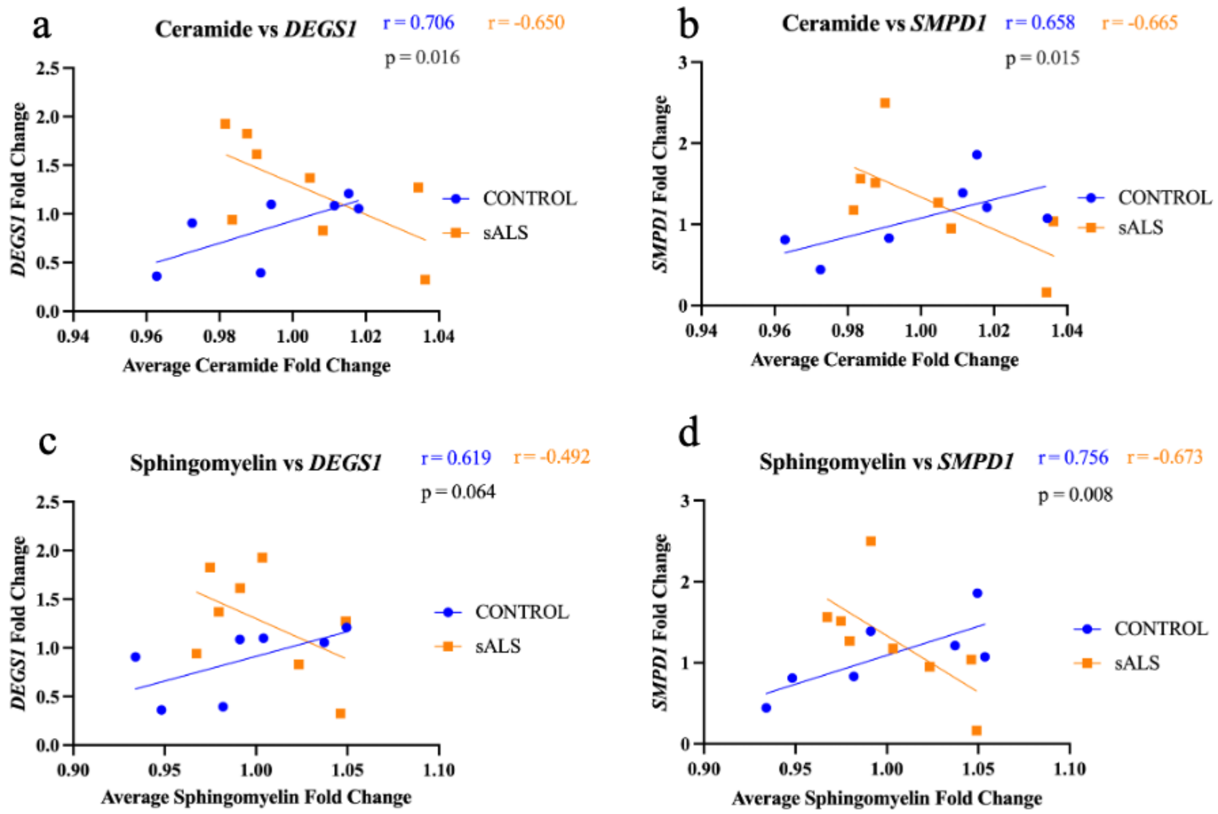

Supplementary Figure S4. There is significant difference in the expression of key lipid metabolizing enzymes relative to their respective lipid species in ALS. The correlation between ceramide levels (expressed as a fold change of the average of all controls) and the gene expression of the enzymes (a) Sphingolipid desaturase (*DEGS1*) and (b) acid sphingomyelinase (SMase, encoded by *SMPD1*) and the correlation between sphingomyelin levels (expressed as a fold change of the average of all controls) and the gene expression of the enzymes (c) Sphingolipid desaturase (*DEGS1*) and (d) acid sphingomyelinase (SMase, encoded by *SMPD1*) in ALS (sALS n=8; orange) and control (n=8; blue).

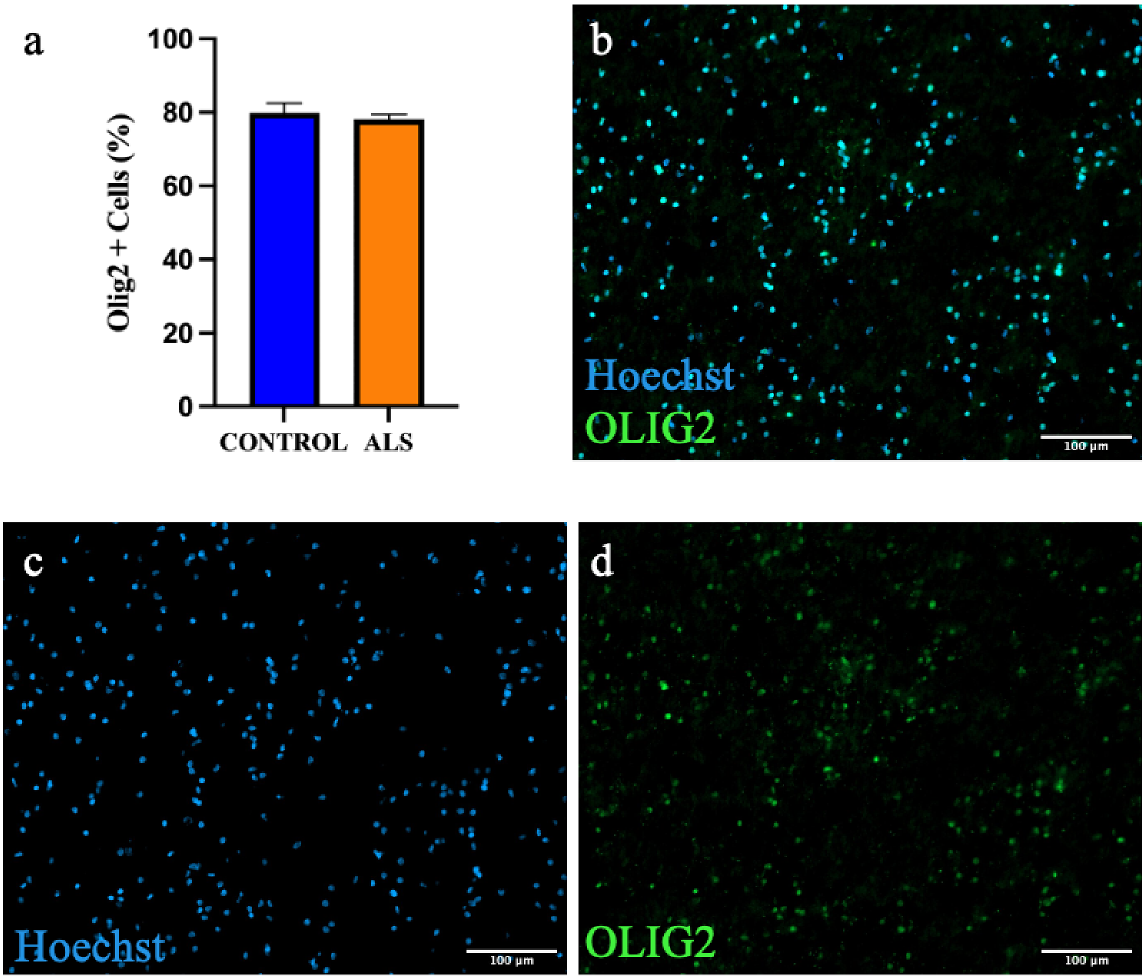

Supplementary Figure S5. There is no difference in the density of OLIG2+ oligodendrocytes in the motor cortex white matter in ALS compared to control. The density of OLIG2+ oligodendrocytes (expressed as a percentage of all Hoechst+ nuclei) in the white matter of the motor cortex is not different in ALS (sALS n=8 and fALS n=6 combined; orange) compared to control (n=8; blue). Representative images of the staining are shown (b) merged; (c) Hoechst alone and (d) OLIG2 alone. Scale bar 100µm.
